# Dataset of long-term compressive strength of concrete with manufactured sand

**DOI:** 10.1016/j.dib.2016.01.065

**Published:** 2016-02-05

**Authors:** Xinxin Ding, Changyong Li, Yangyang Xu, Fenglan Li, Shunbo Zhao

**Affiliations:** a School of Civil Engineering and Communication, North China University of Water Resources and Electric Power, No. 36 Beihuan Road, 450045 Zhengzhou, China; b School of Transportation Engineering, Huanghe Jiaotong University, Wuzhi Yingbin Road, 454950 Zhengzhou, China

**Keywords:** Concrete with manufactured sand(MSC), Long-term compressive strength, Stone powder

## Abstract

This paper presents 186 groups compressive strength tests data of concrete with manufactured sand (MSC) in different curing age and 262 groups compressive strength tests data of MSC at 28 days collected from authors’ experiments and other researches in China. Further interpretation and discussion were described in this issues.

**Specifications table**

**Table:** 

Subject area	Construction and building materials
More specific subject area	Construction materials
Type of data	Tables, figure, text file
How data was acquired	Tests and collection
Data format	Raw and filtered
Experimental factors	Curing ages of 3–388 days and stone powder contents of 3–13%, as well as water-to-cement ratios of 0.32–0.56 were designed in experiments of long-term compressive strength of MSC. Stone powder contents of 0–20% and water-to-cement ratios of 0.30–0.70 were considered in experiments of compressive strength of MSC at 28 days.
Experimental features	Testing the long-term compressive strengths and compressive strengths at 28 days of MSC with different stone powder content at designed curing age in laboratory situation.
Data source location	Zhengzhou City, China, Latitude 34.7568711° and Longitude 113.663221°.
Data accessibility	Data is within this article.

**Value of the data**

• Indicating long-term compressive strength of MSC in laboratory situation.
• Illustrating the long-term compressive strength and compressive strength at 28 days of MSC with different stone powders content included.
• Be useful for comparing compressive strength of MSC with that of concrete made by different aggregates.

**Data**

The long-term compressive strength test data of MSC from authors’ experiments and compressive strength test data of MSC at 28 days collected from authors’ experiments and other researches in China are presented.

## 1 Experimental design, materials and methods

### 1.1 Experimental design

Two experiments have been designed to obtain the long-term compressive strength of MSC [1], [2], [3]. The design details of experiments [1] are presented in Table 1. All samples of compressive strength were designed as cubes in dimension of 150 mm. One group includes three test samples. Samples of experiment [1] were stored at 20±2 °C water for curing, where samples of experiment [2], [3] were left in standard curing box with a temperature of 20±2 °C and humidity of 95–99%.

### 1.2 Materials

Raw materials of experiment [1] were grade P.O. 42.5 ordinary Portland cement, crushed stone mixed in proportion 2:5:2:1 by the series of 5–10 mm, 10–19 mm, 19–26.5 mm and 26.5–31.5 mm, and manufactured sand with different contents of stone powder, as well as tap water and high-performance water reducer. Raw materials of experiment [2], [3] were grade P.O. 42.5 and P.O.32.5 ordinary Portland cement, crushed stone mixed in proportion 1:1 by the series of 5–10 mm and 10–25 mm, and manufactured sand with different contents of stone powder, as well as tap water and high-performance water reducer.

Manufactured sand and crushed stone used in experiments were both crushed from limestone in area of Jiaozuo city, China. Cements were produced by China Tianrui Group Cement Company Limited, Kaifeng.

**Fig. 1:**
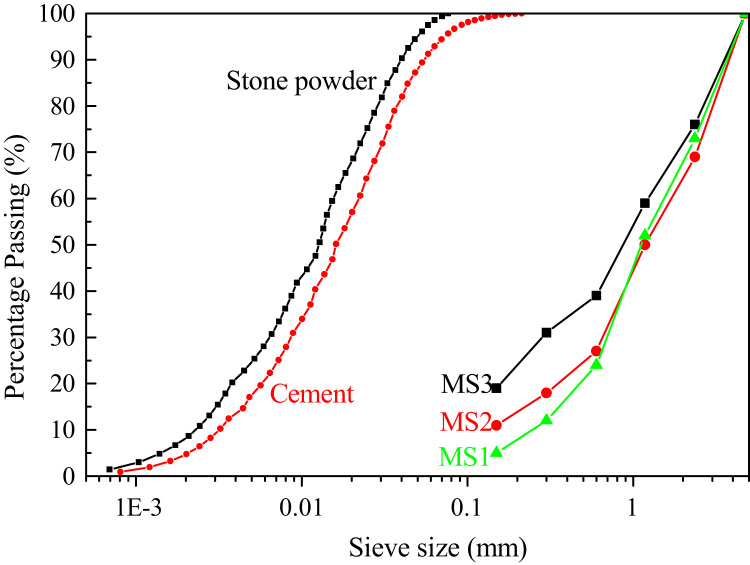
Particle size distribution of stone powder, cement and manufactured sand.

Fig. 1 presents the particle size distribution of stone powder, cement and manufactured sand of experiment [1]. Where MS1, MS2 and MS3 represent manufactured sand with stone powder content as 5%, 9% and 13%, respectively.

### 1.3 Method

Particle size distribution of cement and stone powder was examined by the LS13320 laser diffraction particle size analyzer. Compressive strength of concrete samples was tested on an electro-hydraulic servo universal test machine with maximum load as 2000 kN in accordance with China Standard GB/T 50081-2002 [4] and British Standard BS EN 12390-3-2009 [5].

### 1.4 Effect of stone powder content on long-term compressive strength.

Table 2, Table 3 gives out the long-term compressive strength test data of MSC with different stone powder contents [1], [2], [3].

### 1.5 Compressive strength of MSC at 28 days collected from experiments [6], [7]

Table 4 lists the cubic compressive strength of MSC at 28 days collected from experiments. Where *f*_ce_ represents the cement compressive strength tested in accordance with Standard ISO 679–1989 [8]. The test data of Table 4 has cubic compressive strength at 28 days ranged from 25.0 MPa to 84.6 MPa with water-to-cement ratio as 0.30–0.70, sand ratio of 30–46%, P.O.32.5, P.O42.5 and P.O.52.5 cements in density of 2871–3134 kg/m^3^, coarse aggregate with maximum particle size of 20–31.5 mm, manufactured sand with limestone powder content of 0–20% and fineness modulus of 2.60–3.40.

## Figures and Tables

**Conflict of interest:**
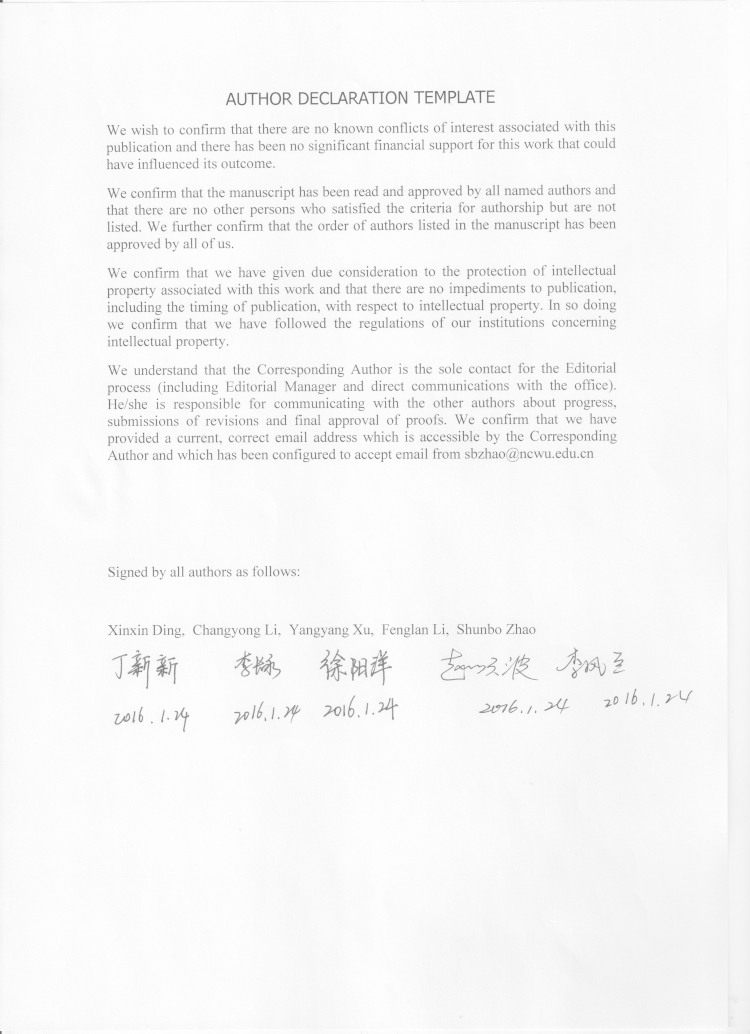


**Table 1:** Experimental design details

Trial no.	Cement type	Water-to cement ratio	Stone powder content (%)	Groups	Desinged curing age (days)
D1	P.O.42.5	0.45	5	20	3, 7, 14, 28, 35, 42, 56, 70, 84, 98, 118, 148, 178,208, 238, 268, 298, 328, 358,388
D2	9
D3	13
C1	0.56	5	12	3, 7, 14, 28, 42, 56, 84, 118, 178, 238, 298, 358
C2	9
C3	13
E1	0.40	5	12	3, 7, 14, 28, 42, 56, 84, 118, 178, 238, 298, 358
E2	9
E3	13
A1	P.O.32.5	0.56	3	9	3(4), 7(8), 14, 28, 56, 90, 120, 150, 180
A2	7
A3	13
B1	P.O.42.5	0.32	3	9	3(4), 7(8), 14, 28, 56, 90, 120, 150, 180
B2	7
B3	13

**Table 2:** Test data of long-term compressive strength in experiment [1].

Curing age (days)	Compressive strength (MPa)								
D1	D2	D3	C1	C2	C3	E1	E2	E3
3	38.4	38	38.1	32.5	28.7	28.5	42.7	39.7	34
7	42.2	42.3	41.7	33.5	34.9	34.7	49.4	49.7	43.6
14	44.9	44.8	44.2	39.7	38.9	37	51.9	51.4	43.8
28	54.2	50.9	51.5	41.2	41.3	40.5	52.9	61.7	57.7
35	51.6	52.3	52.6	–	–	–	–	–	–
42	53.7	53.6	54	42	41.8	42.3	64	62.5	60.6
56	54.2	54.3	56	43.8	42.4	45	65	64	61.2
70	55.3	55	56.5	–	–	–	–	–	–
84	56.5	58.3	57	47.4	48.8	48	66.9	64.6	63
98	58	58.8	57.2	–	–	–	–	–	–
118	59.7	63.7	63	48.9	49.3	48.7	67.3	65	64
148	62.4	64.1	65	–	–	–	–	–	–
178	63.1	64.3	66.2	49.7	51.9	49.9	68	69.6	67.8
208	63.6	65.1	67	–	–	–	–	–	–
238	64.4	67.8	68.7	51.1	55.4	54.5	73	71	68
268	65.4	68.3	69.9	–	–	–	–	–	–
298	67.1	70.8	71.2	51.2	56.2	54.8	74.1	77.9	70.6
328	67.6	71.1	71.4	–	–	–	–	–	–
358	67.8	71.5	72	51.6	56.4	55.1	74.6	78.2	73.1
388	68.2	71.9	72.4	–	–	–	–	–	–

**Table 3:** Test data of long-term compressive strength in experiment [2], [3].

Curing age (days)	Compressive strength (MPa)					
A1	A2	A3	B1	B2	B3
3	–	20.2	23.5	–	–	–
4	27.2	–	–	48	46.5	42
7	–	29.4	28.5	–	–	–
8	31	–	–	51.6	55.9	48.7
14	35.9	39.2	32	58.3	60	60.3
28	40.7	39.5	37.7	60.4	61.2	61
56	47.2	49.3	50.9	67.8	68.1	63.1
90	52	54.5	55.6	69.6	74.4	73.1
120	56.7	57.4	57.5	77.5	78.5	75.9
150	58.5	58.5	59.6	74.1	75.7	75.7
180	60.3	67.4	64.4	82.1	77.9	80.5

**Table 4:** Tests data of cube compressive strength of MSC at 28 days.

*f*_ce_ (MPa)	Stone powder content (%)	Water-to-cement ratio	Sand ratio (%)	Slump (mm)	Compressive strength at 28 days (MPa)
38.2	5/7/10/13/16	0.47	36	16/28/11/17/12	40.7/44.3/45.9/43.4/44.3
47.7	3/5/7	0.32	30	20/11/13	69.6/71.7/74.7
10/13/16	0.32	30	35/20/18	69.8/69.8/70.1
7/7/10/13/16	0.44	32	85/108/75/80/60	57.0/59.6/56.7/57.0/56.2
46.3	5/9/13	0.56	42	135/80/50	42.9/43.8/43.9
5/9/13	0.45	34	70/160/100	48.7/50.1/56.3
5/9/13	0.4	32	150/170/175	55.6/60.7/57.2
5/9/13	0.32	28	50/110/100	70.9/68.1/66.7
52.2	–	0.35/0.40	32/33	–	75.8/67.7
0.45/0.5	35/37	–	64.2/52.0
	0.55	40	–	41.7
49.3	2.7	0.34	30/32/34	45/63/78	59.5/60.1/61.3
2.7	0.34	36/38/40	85/90/120	60.4/59.3/57.5
2.7	0.33/0.34	35	74/82	61.8/60.1
2.7	0.36/0.37	35	100/113	59.1/59.8
2.7	0.33	35	76/83/71	62.0/62.5/61.4
2.7	0.33	35	84/72/86	61.5/61.5/61.8
52.6	0/3/5/7	0.55	42	120/140/150/175	34.6/34.9/35.9/36.4
10/15/20	0.55	42	180/190/160	37.9/38.2/36.5
0/3/5	0.32	42	210/220/220	67.1/69.3/68.3
7/10/15	0.32	42	210/210/205	71.5/74.3/70.6
54.6	5/10/15/20	0.70	41/39/37/35	50/50/45/50	30.0/30.8/30.2/32.6
5/10/15/20	0.65	40/38/36/34	65/45/40/40	34.5/33.2/34.0/34.7
5/10/15/20	0.60	39/37/35/33	70/30/70/70	38.0/37.6/38.1/39.7
5/10/15/20	0.55	38/36/34/32	50/40/50/45	41.7/42.3/43.8/44.1
47.8	5/10/15/20	0.50	40	65/90/65/60	46.7/49.3/45.3/44.8
5/10/15/20	0.50	40	55/65/50/40	46.9/49.3/51.7/48.2
5/10/15/20	0.50	40	60/65/45/30	51.6/46.3/46.9/46.5
0/5/10	0.46	43	80/100/130	43.8/47.2/50.2
15/10/10	0.46	43	95/120/130	45.2/49.9/46.1
1.3/5/7/10/15	0.44	41	30/50/40/30/20	46.2/50.4/46.4/47.5/47.3
10/8.2	0.50/0.46	49/38	195/160	39.5/42.1
9.1/10.3	0.49/0.52	45/42	175/150	38.9/37.1
56.2	1	0.33/0.32	42	120/155	55.2/60.4
45.8	8.2	0.31	34/36	60/45	68.1/70.1
8.2	0.32/0.33	36	65/105	68.1/62.4
8.2	0.33	38	115/105	68.1/64.5
8.2	0.34	40	90	55.3
63.4	3.5	0.30/0.32/0.34	41/42/43	205/210/215	84.6/84.4/82.8
7/10.5/14	0.32	42/40/39	195/210/215	81.5/84.3/87.6
52.3	7	0.42	35	35/45/30/50	49.7/47.7/50.8/47.4
49.0	8.2	0.38	38	85	54.7
45.8	8.2	0.32	36	123	59.3
48.6	8.2	0.32	36	87	63.8
49.2	8.2	0.32	36	145	71.2
55.4	5.8	0.39/0.41/0.43	39	200/200/190	57.1/54.3/50.3
5.8	0.32/0.34	38	195/210	69.0/64.0
5.8	0.36/0.38	38	230/220	65.6/64.3
5.8	0.34/0.36/0.36	37/38/38	165/220/190	61.6/67.7/66.7
5.8	0.35/0.36	39	200/195	65.2/67.7
49.3	5.8	0.32	38	210/220/120	71.0/68.4/66.7
5.8	0.34	38	210/230/170	65.8/67.7/60.6
5.8	0.36	38	180/190/195/200	65.8/65.5/68.4/65.5
5.8	0.37/0.37/0.36	38/38/39	230/185/200	66.1/63.7/66.7
	0.37/0.39/0.37	38	220/205/180	63.4/60.7/64.7
47.8	5/5/10/10	0.46	43	125/125/120/130	43.0/40.2/39.2/45.1
44.8	7/10/15/20	0.48	42	170/180/170/120	52.3/54.0/54.4/55.1
10/15/20	0.5	38	130/125/75	44.8/44.0/44.8
5/10/15/20	0.55	42	155/180/190/160	35.9/35.9/35.9/35.5
56.8	5/7/10/14	0.32	42	225/220/230/230	74.1/76.3/78.9/77.0
45.3	7	0.70/0.6	37/40	10/10	25.0/32.3
0/7	0.5	37	15/15	38.6/38.7
0/7	0.45	39/37	15/20	44.3/43.8
0/7	0.4	38	20/25	45.5/46.5
0/7	0.35	37	10/20	51.5/52.9
63.1	3.5/10.5	0.32	42	210/220	83.5/81.9
61.1	10/15/20	0.65	41.5	35/40/40	37.1/38.1/38.5
44.8	10/15	0.4	45	170/175	49.6/48.6
49.6	–	0.6	46/44/46	200/200/200	29.6/29.1/28.6
–	0.62/0.58	46	180/160	28.2/30.5
